# Cultural adaptation of the speech, spatial and qualities of hearing scale to Colombian Spanish

**DOI:** 10.1016/j.bjorl.2020.02.005

**Published:** 2020-04-10

**Authors:** Diana Carolina Cuéllar Sánchez, Fidel Armando Cañas, Yaná Jinkings de Azevedo, Fayez Bahmad Junior

**Affiliations:** a Universidade de Brasília (UnB), Faculdade de Ciências da Saúde (FCS), Programa de Pós-Graduação em Ciências da Saúde (PPG), Brasília, DF, Brazil; b Universidade de Brasília (UnB), Brasília, DF, Brazil; c Universidade de São Paulo (USP), Faculdade de Medicina (FM), Faculdade de Oftalmologia e Otorrinolaringologia, São Paulo, SP, Brazil; d Instituto Brasileiro de Otorrinolaringologia, Brasilia, DF, Brazil

**Keywords:** Hearing loss, Questionnaire, Portuguese, Spanish

## Abstract

**Introduction:**

The Speech, Spatial and Qualities of Hearing Scale has been widely used to assess the subjective sense of auditory ability, functional hearing loss and the resulting benefit of the hearing correction strategy.

**Objective:**

To translate and culturally adapt the Speech, Spatial and Qualities of Hearing Scale to the Colombian Spanish from Brazilian Portuguese by means of a final version that demonstrates an understanding percentage greater than 85%.

**Methods:**

The study was divided into three phases: in the first one the translation was done, the retro translation and the modifications were defined by the evaluation team, and in the other two, two pilot tests were made to 50 participants: in the first one the understanding of each of the statements that made up the Speech, Spatial and Qualities of Hearing Scale was examined in 25 people and adjustments were made, and in the second, the same procedure was carried out in 25 other individuals but the document was not changed.

**Results:**

It was observed that during the pilot test 1, there was difficulty in questions number 2 of Part 1 (56% understanding), and in 8, 9, 10, 12, 16 and 17 of Part 3 (75%) of understanding), while in others, the degree of understanding was higher than 85%. However, in pilot test 2, understanding was above 85% in all questions. In addition, Cronbach's alpha (0.93) indicated that the items from which the test was constituted measured the same construct and were reliable.

**Conclusion:**

The method used allowed obtaining the version of the Speech, Spatial and Qualities of Hearing Scale in Colombian Spanish with an understanding percentage greater than 85%.

## Introduction

Hearing is one of the most important senses for the human being, not only because it allows one to remain alert in case of any eventuality, but because on a personal level, it grants access to a constant auditory stimulation that generates different sensations, such as those caused by the sound of birds or a stream of water.^1^

The above facilitates the use of hearing in the real world, which includes monitoring the ambient sound, recognizing and locating auditory events, analyzing and controlling one's voice, taking advantage of auditory experiences, and most importantly, perceiving the speech of others and communicating effectively orally.^2^

When auditory decreases occur, changes in the quality of life of people develops,^3^ which is why tools have been developed that examine the daily lives of those who are in these circumstances. Among them are: Handicap Hearing Inventory (HHI), Client Oriented Scale of Improvement (COSI), Open and General Glasgow Benefit Inventory and Entific Medical System QoL.^4^

Also, in 2004 Stuart Gatehouse and William Noble developed the Speech, Spatial and Qualities of Hearing Scale (SSQ), that is currently made up of three parts with 49 questions, the first enquiries about hearing for language (14 questions), the second one enquiries about spatial hearing (17 questions), and the third one, emphasizing the qualities of hearing (18 questions).^5^

The SSQ analyzes the skills to segregate sounds and attend to simultaneous voice flows, as well as the naturalness and clarity in listening yyo or identifying different speakers, pieces, musical instruments and familiar sounds. It is designed to calculate a range of auditory disabilities in several domains, such as language in complex competitive environments, and directional components of distance and movement of spatial hearing.^5^

It has been widely used to assess the subjective sense of auditory ability, functional hearing loss and the resulting benefit of the hearing correction strategy.^6^

Since its creation in English, it has been applied and adapted to other languages, such as Dutch,^7^ Korean,^8^ German,^9^ French,^10^ Persian,^11^ Russian,^12^ Danish, Polish, African, Turkish and Spanish,^13^ however, in the last cited language, formats are available to evaluate children and their parents and teachers.^14^

On the other hand, in its adaptation to Brazilian Portuguese^13^ and to Portugal Portuguese,^15^ it was based on a survey prepared for adults, and originated because the historical contexts of each country produced language differences.^16^

Therefore, the objective of this study is to translate and culturally adapt the SSQ of adults from the Brazilian Portuguese to Colombian Spanish, by means of a final version that demonstrates an understanding percentage greater than 85%, as established by Miranda and Almeida in 2015, in the adaptation they made of the same questionnaire to Brazilian Portuguese.^13^

## Methods

The design was quantitative, descriptive and transversal. This study was approved by the Ethics Committee with the number 14226719.2.0000.0030.

The team was formed by two native Spanish speakers with a Certificate of Proficiency in Portuguese Language (CELPE-BRAS), granted to foreigners with satisfactory performance,^17^ and two native Portuguese speakers with courses in Spanish.

In total 50 people over 18 and under 50 years old, participated. 25 women and 25 men, of Colombian nationality, literate in Spanish, approximately 60% of whom were speech therapists, doctors, biologists, economists, engineers, psychologists, nurses and social workers, while the remaining 40% had finished school.

All signed an informed consent and manifested that they thought they were listening well, and no objective tests were made in this regard because what was sought was to facilitate textual comprehension.

The methodological process was divided into four phases (Table 1), in which all team members intervened.

**Table 1:** Methodological phases.

Phase	1 First version in Spanish	2 Second version in Spanish	3 Final version in Spanish	4 Comparison
Tasks	Translation (Portuguese to Spanish). 2 translators	Pilot test 1 (n = 25)	Pilot test 2 (n = 25)	Final version in Colombian Spanish and Brazilian Portuguese version
	Retro translation (Spanish to Portuguese). 2 other translators			
	Committee of specialists	Committee of specialists		
	Adjustments	Adjustments		

### Phase 1

Two of the researchers did the translation from Portuguese to Spanish and then two others effected the retro translation, that is, the translation from Spanish to Portuguese, to examine the equivalence between languages and to avoid conceptual, of semantic, operational, measurement and functional variations.

Then, it was evaluated by a committee of specialists composed of three speech therapists with experience in the Spanish language area. Necessary modifications were completed and the first version of the document was obtained.

### Phase 2

As in the Brazilian adaptation,^13^ the population was divided into two groups of 25 people each in order to carry out the pilot test 1 to the first and the 2 to the second.

**Figure 1:**
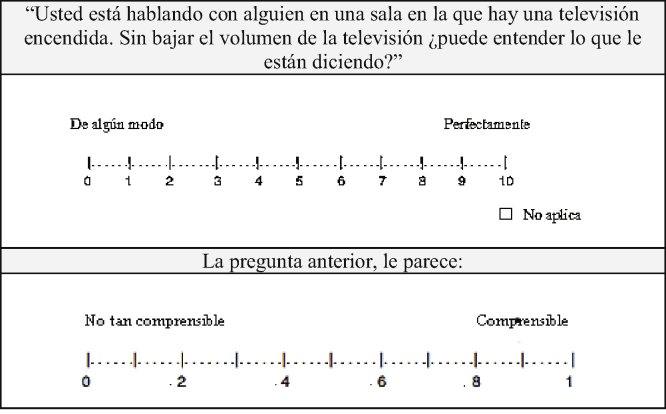
Example of the evaluation format taken from question 1 of part 1 of the SSQ in Colombian Spanish.

At this stage the pilot test number 1 was carried out. The version of the SSQ resulting from Phase 1 was presented to them, and they were asked to read each question to answer how understandable the statement seemed to them in a range of 0–1, where 0 equaled “not so understandable” and 1 to “understandable”, as shown in the example of Fig. 1.

Afterwards, the specialist committee reviewed, the answers were analyzed, the drawbacks were identified, adjustments were executed and the second version was obtained.

### Phase 3

In this part the pilot test 2 was done thanks to the SSQ of the Phase 2. No changes were required, so it was determined that the final version had been achieved.

### Phase 4

The SSQ of Phase 3 was compared with each item of the Brazilian version,^13^ although it was noted that it was valid for the Colombian context and for the same reason it stayed the same.

## Results

In pilot test 1 of Phase 2, it was observed that all had difficulties in questions number 2 of part 1 (56% understanding), and in 8, 9, 10, 12, 16 and 17 of part 3 (75% understanding), because their percentage of understanding was greater than 68% and lower than 84%. It should be noted that in the other questions, they reached an average higher than 85%.

In pilot test 2 of Phase 3, it was noted that all responses were above 85%, which showed that the version achieved was the definitive one.

In Table 2 you can see the data of the averages obtained in the two pilot tests, according to the number of questions that belong to each part of the SSQ, accordingly, there are 10 boxes without values. The shaded areas indicate figures below 85% and the others refer to those that exceeded this percentage.

**Table 2:** Percentages of understanding of SSQ in pilot tests 1 and 2 in Colombian Spanish.

Question (%)	1	2	3	4	5	6	7	8	9	10	11	12	13	14	15	16	17	18
Pilot test 1 ‒ Part 1	92	56	100	100	100	100	100	100	100	100	100	100	100	100	‒	‒	‒	‒
Pilot test 2 ‒ Part 1	100	96	100	100	100	100	100	96	100	100	100	100	100	100	‒	‒	‒	‒
Pilot test 1 ‒ Part 2	100	100	100	100	100	100	100	100	100	100	100	100	100	96	96	92	96	‒
Pilot test 2 ‒ Part 2	100	100	100	100	100	100	100	100	100	96	100	100	100	92	88	96	100	‒
Pilot test 1 ‒ Part 3	100	100	96	100	100	100	100	80	76	72	96	84	100	100	92	71	68	96
Pilot test 2 ‒ Part 3	100	100	100	100	100	100	100	96	96	100	92	100	100	100	100	100	100	100

And in Table 3, a descriptive analysis of the pilot test 2 of Phase 3 is made, where it is evident that the midpoints of part 1, 2 and 3 ranged from 8.0 to 9.8, the standard deviations from 0.50 to 2.50, the minimums from 4 to 9, and all the maximums show a value equal to 10.

**Table 3:** Descriptive analysis of the pilot test 2 of the SSQ in Colombian Spanish.

Part	Question	1	2	3	4	5	6	7	8	9	10	11	12	13	14	15	16	17	18
1	Standard	8.3	9.8	9.0	8.2	8.3	9.1	8.9	8.3	8.6	8.2	8.8	8.1	9.2	8.7	‒	‒	‒	‒
	Deviation	1.40	0.50	1.14	1.26	1.07	1.51	1.38	1.49	1.08	1.50	2.50	1.54	0.99	1.79	‒	‒	‒	‒
	Minimums	6	8	6	6	6	5	6	5	6	5	6	4	7	5	‒	‒	‒	‒
2	Standard	8.0	8.3	8.9	8.5	8.4	8.3	8.1	8.1	8.2	8.0	8.7	8.1	8.8	8.4	8.5	8.2	8.1	‒
	Deviation	1.40	1.43	1.24	1.29	1.29	1.31	1.08	1.29	1.53	1.58	1.54	1.54	1.29	1.71	1.42	1.40	1.36	‒
	Minimums	5	5	6	6	6	5	6	5	5	5	5	5	6	5	6	5	5	‒
3	Standard	9.0	8.2	8.8	9.1	9.6	9.0	8.0	9.0	9.0	8.8	9.0	9.0	8.3	9.0	8.4	8.3	8.9	8.0
	Deviation	0.87	1.16	1.01	1.01	0.50	1.08	1.40	1.17	1.04	1.22	1.08	1.21	1.44	1.38	1.26	1.50	0.88	1.44
	Minimums	8	5	7	6	9	6	5	5	7	5	6	7	5	6	5	5	7	5

In the same manner, Cronbach's alpha was found, which is a method that calculates internal consistency to determine the reliability of a measuring instrument,^18^ and, a value of 0.930 was obtained, indicating that the items for which the scale was composed measured the same construct and were reliable (Table 4).

**Table 4:** Measures of internal consistency of the SSQ in Colombian Spanish.

Part	Question nº	Cronbach Alpha
1	14	0.731
2	17	0.852
3	18	0.881
Total	49	0.930

## Discussion

In pilot test 1, Miranda and Almeida found comprehension averages lower than 85%, in question 14 of part 2 and in 5 of 3,^13^ while in this study they were presented in 2 of Part 1, and in 8, 9, 10, 12, 16 and 17 of 3. In spite of that, in both cases the other questions attained an average of understanding superior to the 85%.

On the other hand, in Portugal the participants expressed doubts in 1, 2, 3, 4, 6, 8 and 9 of Part 1, in 10, 11, 12, 13, 14 and 16 of part 2 and in 7 and 10 of Part 3.^15^

In pilot test 2, in Brazil they achieved 91.6% understanding in all questions^13^ and in Colombia exceeded 85%; this means that the improvements made by both groups after pilot test 1 were effective.

For Brazilians the midpoints of the parties 1, 2 and 3 oscillated between 5.8 and 9.7, the standard deviations from 0.57 to 3.32 and the minimums from 0 to 8,^13^ instead for Colombians, the midpoints were between 8.0 and 9.8, the standard deviations from 0.50 to 2.50 and the minimums from 4 to 9. In the two investigations all the maximums were equal to 10, except for the first ones because in 14 of part 1 they reached a 9.^13^

Taking into account the 49 questions that constituted the test, the Cronbach's alpha in the Brazilian,^13^ Colombian, French^10^ and Iranian^11^ versions was greater than 0.9, which indicated that the work carried out had yielded reliable results.

As can be seen, the methodology allowed the adaptations to be made in each of the countries in question, because it is a procedure that works and could be used in other regions.

Even so, when applying the test, some words caused errors of interpretation because they did not help understanding the meaning of the inquiries, as was the case of question 2 of part 1, which initially referred to a “carpet (alfombra in Spanish)”, or in questions 8, 9, 10, 12, 16 and 17 of Part 3, where the response options decreased the average comprehension.

**Figure 2:**
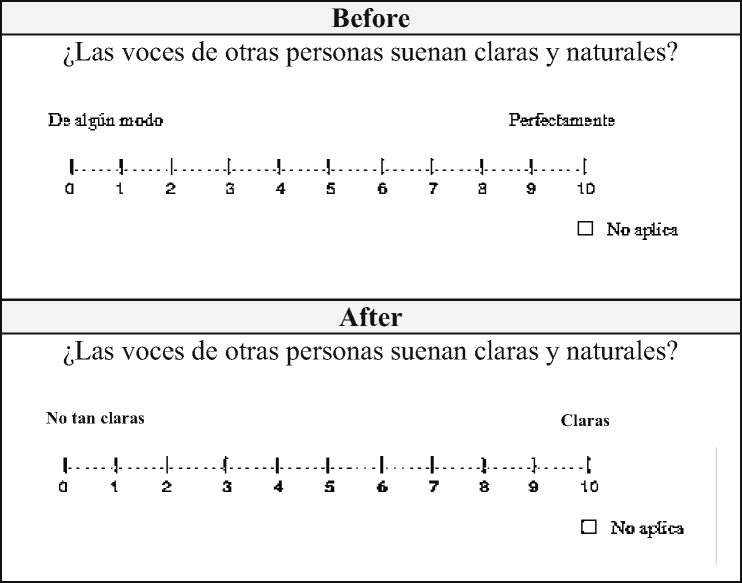
Example of the evaluation format taken from question 10 of part 3 of the SSQ in Colombian Spanish.

For example, in question number 10 of Part 3 (do other people's voices sound clear and natural) and in the answer: somehow vs. perfectly; this caused confusion because there seemed to be no coherence between the question and the answer, thus it was modified to: not so clear vs. clear (Fig. 2). In the other questions in which difficulty was found similar changes were made.

As in the adaptation of European Portuguese,^15^ several people stated that the test was very extensive, so that the size of the letter was reduced and the design was reformed so that it would be visually shorter. These observations were also made by some researchers in Brazil^19^ and in France,^20^ which is why they proposed a version with fewer questions.

There is no doubt that this instrument allows an effective evaluation before, during and after the auditory rehabilitation, and that its versatility means that it can be applied to people who use different types of single or bilateral support products.^15^ For this reason, it is expected that in this way a better follow-up will be done to the users who are in therapy, and the use of the questionnaire in clinical practice be promoted.^21^

As a step to follow, it has elevated its adaptation and pediatric validation and its validation for adults, both in long format and in short format, because this questionnaire needs a standard intercultural adaptation to be able to be compared in several languages and countries.^22^

## Conclusion

The method used to carry out the translation and adaptation of the questionnaire from Brazilian Portuguese resulted in the version of the SSQ in Colombian Spanish (Supplementary material 1), with an understanding percentage greater than 85%.

## Conflicts of interest

The authors declare no conflicts of interest.
