# Supplementary material for: Cultural adaptation of the speech, spatial and qualities of hearing scale to Colombian Spanish
Source: Braz J Otorhinolaryngol. 2020 Apr 10;88(1):4–8. doi: 10.1016/j.bjorl.2020.02.005 (PMC9422663; doi:10.1016/j.bjorl.2020.02.005)
Supplement: Supplementary file 1 [file mmc1.pdf]

Apendix. Adapted version of the SSQ to Colombian Spanish.

Orientaciones sobre cómo responder las preguntas

Los siguientes interrogantes abordan aspectos de su capacidad y experiencia de oír y escuchar en diferentes situaciones.

Para cada pregunta, marque una (X) en cualquier lugar de la escala presentada, la cual varía de 0 a 10. Colocar una (X) en el 10, significa que usted sería perfectamente capaz de hacer o experimentar lo que está descrito. Poner una (X) en el 0, quiere decir que usted sería incapaz de hacer o experimentar lo que se describe.

Por ejemplo, la pregunta 1 se refiere al momento en el cual se sostiene una conversación con alguien mientras la televisión está encendida. Si usted es perfectamente capaz de hacerlo, entonces debe señalar el final de la escala a la derecha. Si usted podría acompañar cerca de la mitad de la conversación en esa situación, debe marcar en el punto medio de la escala y así sucesivamente.

Esperamos que todas las cuestiones sean relevantes y cotidianas, pero si una pregunta describe una situación que no se aplica a usted, marque una (X) en "no aplica"; también escriba el motivo al lado.

Por favor, responda las siguientes preguntas y continúe con el cuestionario sobre su audición.

Nombre:

Fecha:

Edad:

Marque una de las siguientes opciones:

☐ No tengo prótesis auditivas

☐ Uso una prótesis auditiva (Oído izquierdo)

☐ Uso una prótesis auditiva (Oído derecho)

☐ Uso dos prótesis auditivas (Ambas orejas)

- Si usted utiliza prótesis auditivas ¿desde hace cuánto tiempo lo hace?

  ______ años______ meses o______ semanas

- Si usted tiene dos prótesis auditivas y las ha utilizado por períodos de tiempo diferentes, por favor, anote la información sobre cada una de ellas.

Parte 1: Audición para el lenguaje	
**1.** Usted está hablando con alguien en una sala en la que hay una televisión encendida. Sin bajar el volumen de la televisión ¿puede entender lo que le están diciendo?	De algún modo Perfectamente<br>0 1 2 3 4 5 6 7 8 9 10<br>☐ No aplica
**2.** Usted está hablando con alguien en una sala silenciosa ¿puede entender lo que le están diciendo?	De algún modo Perfectamente<br>0 1 2 3 4 5 6 7 8 9 10<br>☐ No aplica
**3.** Usted está en un grupo de más o menos 5 personas sentadas alrededor de una mesa. El lugar es silencioso, estas personas están hablando y usted puede ver a cada uno del grupo ¿logra comprender la conversación?	De algún modo Perfectamente<br>0 1 2 3 4 5 6 7 8 9 10<br>☐ No aplica
**4.** Usted está en un grupo de más o menos 5 personas, en un restaurante muy concurrido, y puede ver a cada uno del grupo ¿usted logra comprender la conversación?	De algún modo Perfectamente<br>0 1 2 3 4 5 6 7 8 9 10<br>☐ No aplica
**5.** Usted está hablando con alguien. Hay un ruido continuo en el medio ambiente, como de un ventilador o de un chorro de agua ¿usted logra comprender la conversación?	De algún modo Perfectamente<br>0 1 2 3 4 5 6 7 8 9 10<br>☐ No aplica
**6.** Usted está en un grupo más o menos de 5 personas en un restaurante concurrido. Usted NO puede ver a todos los del grupo ¿logra comprender la conversación?	De algún modo Perfectamente<br>0 1 2 3 4 5 6 7 8 9 10<br>☐ No aplica
**7.** Usted está hablando con alguien en un lugar en donde hay mucho eco, como en una iglesia o en una estación de tren ¿logra entender lo que le están diciendo?	De algún modo Perfectamente<br>0 1 2 3 4 5 6 7 8 9 10<br>☐ No aplica
**8.** ¿Usted puede conversar con alguien que tiene el mismo tono de voz de otra persona que también está hablando?	De algún modo Perfectamente<br>0 1 2 3 4 5 6 7 8 9 10<br>☐ No aplica

**9.** ¿Usted puede conversar con alguien que tiene el tono de voz diferente al de otra persona que también está hablando?	De algún modo Perfectamente<br>0 1 2 3 4 5 6 7 8 9 10<br>☐ No aplica
**10.** Usted está oyendo a alguien que le está hablando y al mismo tiempo intenta oír las noticias de la televisión ¿puede entender lo que dicen los dos?	De algún modo Perfectamente<br>0 1 2 3 4 5 6 7 8 9 10<br>☐ No aplica
**11.** Usted está conversando con alguien en una sala en la que hay muchas personas hablando al mismo tiempo ¿logra entender lo que le está diciendo?	De algún modo Perfectamente<br>0 1 2 3 4 5 6 7 8 9 10<br>☐ No aplica
**12.** Usted está en un grupo y la charla cambia de una persona a otra ¿puede comprender con facilidad la conversación sin perder el inicio de lo que cada una dice?	De algún modo Perfectamente<br>0 1 2 3 4 5 6 7 8 9 10<br>☐ No aplica
**13.** ¿Usted puede tener una conversación por teléfono sin dificultad?	De algún modo Perfectamente<br>0 1 2 3 4 5 6 7 8 9 10<br>☐ No aplica
**14.** Usted está hablando por teléfono y alguien que está cerca empieza a hablar ¿puede entender lo que dicen ambos?	De algún modo Perfectamente<br>0 1 2 3 4 5 6 7 8 9 10<br>☐ No aplica
Parte 2: Audición espacial	
**1.** Usted está al aire libre en un lugar desconocido y oye a alguien usando una trituradora, pero NO puede ver en dónde está ¿usted puede decir inmediatamente de dónde viene el sonido?	De algún modo Perfectamente<br>0 1 2 3 4 5 6 7 8 9 10<br>☐ No aplica
**2.** Usted está sentado alrededor de una mesa o en una reunión con varias personas, pero NO puede ver a todas ¿usted puede decir dónde está cada una tan pronto como empieza a hablar?	De algún modo Perfectamente<br>0 1 2 3 4 5 6 7 8 9 10<br>☐ No aplica
**3.** Usted está sentado entre dos personas y una de ellas comienza a hablar ¿usted puede decir inmediatamente si es la de su derecha o la de su izquierda, sin tener que mirar?	De algún modo Perfectamente<br>0 1 2 3 4 5 6 7 8 9 10<br>☐ No aplica

**4.** Usted está en una casa desconocida, está en silencio y oye una puerta golpear bien fuerte ¿usted puede decir inmediatamente de donde vino ese sonido?	De algún modo Perfectamente<br>0 1 2 3 4 5 6 7 8 9 10<br>☐ No aplica
**5.** Usted está en la escalera de un edificio con pisos por encima y por debajo, y oye sonidos de otro piso ¿usted puede decir rápidamente de dónde viene el sonido?	De algún modo Perfectamente<br>0 1 2 3 4 5 6 7 8 9 10<br>☐ No aplica
**6.** Usted está al aire libre y un perro late bien fuerte ¿usted puede decir inmediatamente en dónde se encuentra sin necesidad de mirar?	De algún modo Perfectamente<br>0 1 2 3 4 5 6 7 8 9 10<br>☐ No aplica
**7.** Usted está en la acera de una calle muy transitada ¿puede identificar de inmediato en qué dirección viene un autobús o camión antes de verlo?	De algún modo Perfectamente<br>0 1 2 3 4 5 6 7 8 9 10<br>☐ No aplica
**8.** En la calle ¿usted puede decir si alguien está lejos, por el sonido de su voz o de sus pasos?	De algún modo Perfectamente<br>0 1 2 3 4 5 6 7 8 9 10<br>☐ No aplica
**9.** ¿Usted puede decir cuánto está lejos un autobús o un camión, a partir de su sonido?	De algún modo Perfectamente<br>0 1 2 3 4 5 6 7 8 9 10<br>☐ No aplica
**10.** Usted puede decir a partir del sonido ¿cuál es la dirección del movimiento de un autobús o camión, por ejemplo, de izquierda a derecha o de derecha a izquierda?	De algún modo Perfectamente<br>0 1 2 3 4 5 6 7 8 9 10<br>☐ No aplica
**11.** ¿Usted puede decir por el sonido de la voz o de los pasos, en qué dirección una persona se está moviendo, por ejemplo, de izquierda a derecha o de derecha a izquierda?	De algún modo Perfectamente<br>0 1 2 3 4 5 6 7 8 9 10<br>☐ No aplica
**12.** ¿Usted puede decir por el sonido de la voz o de los pasos si una persona está viniendo en su dirección o alejándose?	De algún modo Perfectamente<br>0 1 2 3 4 5 6 7 8 9 10<br>☐ No aplica

**13.** ¿Usted puede decir a partir del sonido si un autobús o camión está viniendo en su dirección o alejándose?	De algún modo … Perfectamente<br>0 1 2 3 4 5 6 7 8 9 10<br>☐ No aplica
**14.** ¿Los sonidos que usted oye parecen estar dentro de su cabeza más que fuera de ella (en el mundo exterior)?	Dentro de la cabeza … Fuera de ella<br>0 1 2 3 4 5 6 7 8 9 10<br>☐ No aplica
**15.** Los sonidos de las personas o de las cosas que usted oye pero NO puede ver de inmediato ¿acaban por estar más cerca de lo esperado cuando usted las ve?	Muy cerca … No tan cerca<br>0 1 2 3 4 5 6 7 8 9 10<br>☐ No aplica
**16.** Los sonidos de las personas o de las cosas que usted oye pero NO puede ver de inmediato ¿acaban por estar más lejos de lo esperado cuando usted las ve?	Muy lejos … No tan lejos<br>0 1 2 3 4 5 6 7 8 9 10<br>☐ No aplica
**17.** ¿Usted tiene la impresión de que los sonidos están exactamente donde usted esperaría que estuvieran?	De algún modo … Perfectamente<br>0 1 2 3 4 5 6 7 8 9 10<br>☐ No aplica
Parte 3: Cualidades de la audición	
**1.** Piense en cuando usted oye dos cosas al mismo tiempo, por ejemplo, agua corriendo en una fuente y una radio encendida ¿usted tiene la impresión de que estos sonidos suenan separados el uno del otro?	De algún modo … Perfectamente<br>0 1 2 3 4 5 6 7 8 9 10<br>☐ No aplica
**2.** Cuando usted oye más de un sonido al mismo tiempo ¿tiene la impresión de que parece ser un sonido único mezclado?	Mezclado … No mezclado<br>0 1 2 3 4 5 6 7 8 9 10<br>☐ No aplica
**3.** Usted está en una habitación, tiene una radio con música, y alguien en la sala está hablando ¿usted puede oír la voz como algo separado de la música?	De algún modo … Perfectamente<br>0 1 2 3 4 5 6 7 8 9 10<br>☐ No aplica
**4.** ¿Usted encuentra fácil reconocer a las personas conocidas por el sonido de su voz?	De algún modo … Perfectamente<br>0 1 2 3 4 5 6 7 8 9 10<br>☐ No aplica

**5.** ¿Usted encuentra fácil distinguir las diferentes canciones que usted conoce?	De algún modo — Perfectamente<br>0 1 2 3 4 5 6 7 8 9 10<br>☐ No aplica
**6.** ¿Usted puede decir la diferencia entre sonidos, por ejemplo, de un coche y de un autobús, o de agua hirviendo en una olla y de alimentos fritándose en una sartén?	De algún modo — Perfectamente<br>0 1 2 3 4 5 6 7 8 9 10<br>☐ No aplica
**7.** Cuando usted oye música ¿logra distinguir qué instrumentos están tocando?	De algún modo — Perfectamente<br>0 1 2 3 4 5 6 7 8 9 10<br>☐ No aplica
**8.** Cuando usted oye música ¿el sonido es claro y natural?	No tan claro — Claro<br>0 1 2 3 4 5 6 7 8 9 10<br>☐ No aplica
**9.** ¿Los sonidos del día a día que usted logra escuchar con facilidad son claros (no turbios)?	Turbios — Claros<br>0 1 2 3 4 5 6 7 8 9 10<br>☐ No aplica
**10.** ¿Las voces de otras personas suenan claras y naturales?	No tan claras — Claras<br>0 1 2 3 4 5 6 7 8 9 10<br>☐ No aplica
**11.** ¿Los sonidos del día a día que usted oye parecen tener una calidad artificial o natural?	Poco natural — Natural<br>0 1 2 3 4 5 6 7 8 9 10<br>☐ No aplica
**12.** ¿El sonido de su propia voz le parece natural?	Poco natural — Natural<br>0 1 2 3 4 5 6 7 8 9 10<br>☐ No aplica
**13.** ¿Usted puede juzgar fácilmente el humor de otra persona por el sonido de su voz?	De algún modo — Perfectamente<br>0 1 2 3 4 5 6 7 8 9 10<br>☐ No aplica
**14.** ¿Usted tiene que concentrarse mucho cuando está escuchando alguna cosa o a alguien?	Necesita concentrarse mucho — No necesita concentrarse<br>0 1 2 3 4 5 6 7 8 9 10<br>☐ No aplica

**15.** ¿Usted tiene que esforzarse mucho para escuchar lo que se está diciendo en una conversación?	Mucho esfuerzo — Ningún esfuerzo<br>0 1 2 3 4 5 6 7 8 9 10<br>☐ No aplica
**16.** Cuando usted es el conductor de un coche ¿puede escuchar fácilmente lo que está hablando la persona que va sentada a su lado?	No tan fácil — Fácilmente<br>0 1 2 3 4 5 6 7 8 9 10<br>☐ No aplica
**17.** Cuando usted es pasajero y va sentado al lado del conductor ¿puede oír fácilmente lo que le está hablando?	No tan fácil — Fácilmente<br>0 1 2 3 4 5 6 7 8 9 10<br>☐ No aplica
**18.** ¿Usted puede ignorar fácilmente otros sonidos al intentar escuchar alguna cosa?	Difícil ignorar — Fácil ignorar<br>0 1 2 3 4 5 6 7 8 9 10<br>☐ No aplica
